# Pheochromocytoma: A Case Report

**DOI:** 10.7759/cureus.31409

**Published:** 2022-11-12

**Authors:** Eulália Antunes, Joana Lopes, Isabel Silva, Vera Fernandes

**Affiliations:** 1 Internal Medicine Department, Hospital de Braga, Braga, PRT; 2 Endocrinology Department, Hospital de Braga, Braga, PRT

**Keywords:** catecholamines secretion, phenoxybenzamine, pheochromocytoma, suprarenal nodule, hypertension

## Abstract

Pheochromocytomas are rare tumors located in the adrenal medulla, that derives from the chromaffin cells and produce catecholamines. They are an uncommon cause of hypertension, and only 50% of the patients present symptoms compatible with this pathology. Here we describe the case of a 70-year-old woman with a history of anxiety, hypertension and palpitation, who had an unspecified nodule in the right adrenal gland. Laboratory studies revealed an elevated urinary metanephrines secretion. A diagnosis of pheochromocytoma was made and an adrenalectomy was performed. Our aim is to highlight the diagnosis of this rare tumor and how its early management can prevent morbidity and mortality.

## Introduction

The global prevalence of hypertension (defined as blood pressure higher than 140/90 mmHg) is high all over the world, and it is responsible for great cardiovascular morbidity and mortality. However, 3-10% is due to secondary hypertension, with a potential of cure, so it is important to consider secondary causes, if the clinic and course of the disease suggest it [1].

Pheochromocytoma is a rare neoplasm with an incidence of 1-4/10^6^ population/year. This tumor is known for causing hypertension, however, it is an uncommon cause of hypertension, and occurs in less than 1% of hypertensive patients [2]. This tumor derives from the chromaffin cells of the embryonic neural crest, which produce catecholamines. These cells are mainly located in the adrenal medulla, but they can also appear in extra-adrenal locations, called paragangliomas. Clinical presentation varies according to location and degree of catecholamine secretion, and includes the classic triad of headaches, palpitations, and profuse sweating [2,3]. The diagnosis of pheochromocytoma requires excessive release of catecholamines and anatomical documentation of the tumor. Surgical resection of pheochromocytoma is the keystone of therapy, following which hypertension can be cured or easily controlled [3].

## Case presentation

**Figure 1:**
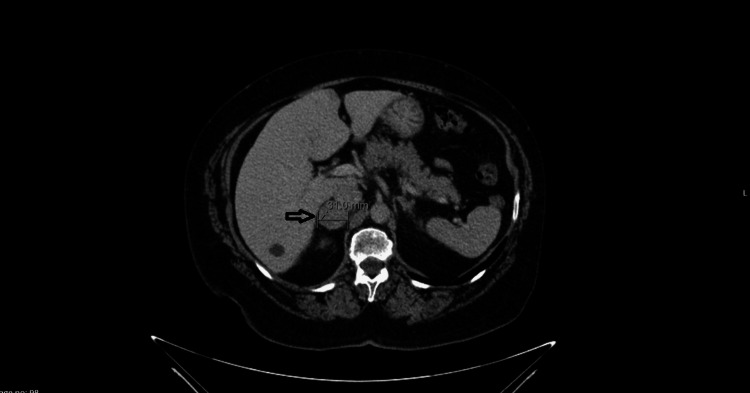
Abdominal CT scan showing a homogeneous lesion, with 31 mm of diameter, in the right adrenal gland

A 70-year-old woman was referred by her family physician because of recurrent abdominal pain and the evidence of a suprarenal nodule with 31 mm of diameter in an abdominal CT scan (Figure 1). She had a history of anxiety, hypertension, dyslipidemia, obesity and palpitation, for which she was followed up in a cardiology consultation. The patient also reported tremor and a lack of strength in the lower limbs. On physical examination, she had pale skin and no other change - no palpable masses or heart or abdominal murmurs. Laboratory studies revealed an elevated urinary metanephrines secretion of 1080 ug/24h (normal value under 341 ug/24h) and also an elevated urinary normetanephrine of 734 ug/24h (normal value under 444 ug/24h) (Table 1). The remaining analytical study did not reveal any changes. A diagnosis of pheochromocytoma was made. A PET-SCAN was requested which excluded the presence of metastatic disease or multiple chromaffin tumors. Treatment was guided by endocrinology and general surgery, in order to achieve alpha and beta-adrenergic blockade prior to surgery. Phenoxybenzamine (10 mg per day) was initiated 10 days before surgery. Laparotomic excision of the right adrenal gland was successful. Histological analysis confirmed the diagnosis of pheochromocytoma, without invasion of peri-adrenal adipose tissue. In the post-operative stage, the patient was admitted to an intermediate care unit, but no hypotension or hypoglycemia was documented. The results of the genetic study were negative. Post-op urinary metanephrine and normetanephrine were within the normal range (Table 1). Three months after the adrenalectomy, ambulatory blood pressure monitoring was normal and the patient denied previous symptoms.

**Table 1:** Analytical values found in the first assay and six months after surgery

	Urinary metanephrines	
	Metanephrine	Normetanephrine
Normal range	< 341 ug/24h	< 444 ug/24h
Values found in the first assay	1080 ug/24h	736 ug/24h
Values found after surgery	31 ug/24h	299 ug/24h

## Discussion

Catecholamine-secreting tumors are a rare neoplasm, occurring in approximately 0.1 to 1% of hypertensive patients [2]. Pheochromocytomas are most common in the fourth to fifth decade, although they can occur at any age. They are equally common in females and males [4].

The clinical presentation varies, alternating from an adrenal incidentaloma to hypertensive crises with associated cerebrovascular or cardiac complications. Only 4% of adrenal masses incidentally found are known to be pheochromocytomas, the most common are benign adenomas [2]. In the present case, the mass had characteristics more similar to an adenoma - homogeneous density, a diameter less than 4 cm and unilateral location - however, together with the symptoms, the diagnosis of pheochromocytoma seemed more likely. Symptoms are present in approximately 50% of patients and, when present, they are typically paroxysmal and are attributable to the excess of catecholamines released by tumors - epinephrine, norepinephrine or dopamine [2,5].

The diagnosis of this pathology requires both proof of excessive release of catecholamines and anatomical documentation of the tumor [3]. Once a pheochromocytoma has been identified a genetic test must be performed, because 35-40% of diagnosed patients have a germline mutation, which associates with an added risk of transmission and malignancy. There are several familial disorders associated with adrenal pheochromocytoma, the most common are: von Hippel-Lindau syndrome (VHL), multiple endocrine neoplasia type 2 (MEN2) and neurofibromatosis type 1 (NF1) [3]. Although most tumors are benign, about 10% of pheochromocytomas are malignant, and the only reliable clue to the presence of malignant pheochromocytoma is a local invasion into surrounding tissues and organs or distant metastases documented on nuclear imaging [3,6].

Surgery is the treatment of choice for these tumors, curable for more than 90% of patients. The preoperative preparation is essential to reduce the perioperative morbidity and mortality in these patients [3,5]. Alpha-blockade, fluid and salt intake are recommended by the Endocrine Society for patients undergoing pheochromocytoma resection, to minimize hemodynamic instability during tumor manipulation [5]. Phenoxybenzamine is the election drug used. It is initiated at doses of 10 mg every 6-12h and increased, if clinically needed, to 30-40 mg every 6h to a maximum of 240mg/day [5]. The beta-antagonist should be administered to avoid symptoms, like tachycardia, after alpha-adrenergic blockade has been effective in normalizing blood pressure [3]. After the surgery, hypotension can occur in 20-70%. The sudden catecholamine withdrawal, after tumor removal, also leads to rebound hyperinsulinemia which along with already depleted glycogen stores can lead to severe hypoglycemia in the postoperative period. Thus, monitoring arterial pressure and blood sugar are mandatory after surgery [5].

## Conclusions

Pheochromocytomas are rare neuroendocrine tumors responsible for less than 1% of hypertensive cases. Only 50% of individuals will present symptoms compatible with this tumor and, in most cases, the symptoms will be paroxysmal. Surgery is curative for pheochromocytomas but long-term surveillance is necessary.

## References

[1] Barroso WKS, Rodrigues CIS, Bortolotto LA (2021). Brazilian guidelines on arterial hypertension - 2020. Arch Bras. Cardiol.

[2] Soltani A, Pourian M, Davani BM (2016). Does this patient have pheochromocytoma? A systematic review of clinical signs and symptoms. J Diabetes Metab Disord.

[3] Neumann HP, Young WF Jr, Eng C (2019). Pheochromocytoma and paraganglioma. N Engl J Med.

[4] Guerrero MA, Schreinemakers JM, Vriens MR (2009). Clinical spectrum of pheochromocytoma. J Am Coll Surg.

[5] Ramachandran R, Rewari V (2017). Current perioperative management of pheochromocytomas. Indian J Urol.

[6] Martins R, Bugalho MJ (2014). Paragangliomas/pheochromocytomas: clinically oriented genetic testing. Int J Endocrinol.

